# Long-Term Outcome of the Anomalous Origin of the Left Coronary Artery From the Pulmonary Artery (ALCAPA) in Children After Cardiac Surgery: A Single-Center Experience

**DOI:** 10.7759/cureus.11829

**Published:** 2020-12-01

**Authors:** Muna Ismail, Abdulraouf Jijeh, Rathath M Alhuwaymil, Raneem Alahmari, Rawan Alshahrani, Reem Almutairi, Fahad Habshan, Ghassan A Shaath

**Affiliations:** 1 Cardiac Sciences Department, Pediatric Cardiology, King Abdulaziz Cardiac Center, Ministry of the National Guard - Health Affairs, Riyadh, SAU; 2 Cardiac Sciences Department, Pediatric Cardiac Intensive Care Unit, Ministry of National Guard - Health Affairs, Riyadh, SAU; 3 Echocardiographic Technology, College of Applied Medical Sciences King Saud bin Abdulaziz University for Health Sciences, Riyadh, SAU; 4 King Abdulaziz Cardiac Center, Pediatric Cardiology, King Abdulaziz Medical City Riyadh, Riyadh, SAU; 5 Cardiac Sciences Department, Pediatric Cardiac Intensive Care Unit, King Abdulaziz Cardiac Center. Ministry of the National Guard - Health Affairs, Riyadh, SAU

**Keywords:** anomalous origin of the left coronary artery from pulmonary artery, children, ejection fraction, fractional shortening, echocardiography

## Abstract

Background

The anomalous origin of the left coronary artery from the pulmonary artery (ALCAPA) is a rare congenital coronary artery anomaly. It induces left ventricular (LV) dysfunction and mitral valve regurgitation (MR). If untreated, survival beyond infancy is rare. The aim of our study was to analyze the outcome in children with ALCAPA after cardiac surgery.

Methods

We retrospectively reviewed all patients who were diagnosed at our institution with ALCAPA and underwent surgical repair from 1999 to the end of 2018 (for 20 years). We followed LV dimensions, function, the progress of MV regurgitation, and the somatic growth of children after surgical repair.

Results

Twenty-nine patients underwent ALCAPA repair while 15 (52%) patients were male. The median age at surgical repair was 5.3 (IQR: 3.8-7.4) months and the mean weight was 5.5±2 kg. Surgical repair was performed in form of coronary reimplantation in 26 (90%) patients and Takeuchi repair in three (10%) patients. Intensive care unit (ICU) stay was eight (IQR: 6-17) days and hospital stay was 15 (IQR: 12-21) days. The follow-up duration was 5±3.6 years. Echocardiographic parameters started to improve by six weeks after the repair, and they normalized by one year. At the time of surgery ejection fraction (EF) was 34±17%, fractional shortening (FS) was 15±10%, and LV inner diameter in diastole (LVIDD) z score was 5.7±2.8. These parameters improved by one year after surgery to 66±7%, 34±6%, and 0±1.3, respectively. However, somatic growth started to improve six months after surgical repair. MR was moderate to severe in seven (24%) patients at the time of surgery and regressed to no more moderate nor severe MR at the last follow-up. None of the 29 patients died.

Conclusions

LV systolic function and dimensions start to improve by six weeks after surgery and reach normal values by one year. MR regresses without intervention in correspondence with the regression of LV dimensional parameters. The somatic growth of children improves six months after repair.

## Introduction

Although the anatomy of the anomalous origin of the left coronary artery from the pulmonary artery (ALCAPA) was first described in 1911 by Abrikossoff [1], the first comprehensive clinical description of the disease was provided in 1933 by Bland, White, and Garland. They had described in their report a three-month-old boy with ALCAPA; since then, it was described as Bland-White-Garland syndrome [2-4]. This cardiac defect is a rare form of coronary artery anomaly that occurs in one in 250 of all congenitally malformed hearts, with an overall incidence of approximately one in 300,000 live births with 70% as boys and leads to death in the first year of life in 90% of unoperated cases. Rarely, ALCAPA is associated with other cardiac anomalies such as coarctation of the aorta (COA), atrial septal defect (ASD), ventricular septal defect (VSD), and patent ductus arteriosus (PDA) [3-5].

Fetuses with ALCAPA remain asymptomatic because the diastolic pressure in the pulmonary artery and aorta are similar during prenatal circulation. When the pulmonary vascular resistance starts to drop after birth, symptoms start to appear in most infants due to a reversal flow through the left coronary artery. This leads to coronary artery steal and further progression of myocardial ischemia. Mitral valve regurgitation (MR) occurs in the disease process secondary to ventricular dilatation and papillary muscle ischemia [4,6-7].

Several surgical techniques have been described for ALCAPA repair, including coronary artery re-implantation to the aorta, intrapulmonary artery baffle (Takeuchi procedure), bypass grafting, and ligation of the anomalous coronary artery. Mitral valve (MV) competency improves during follow-up in correspondence with the improvement of ventricular dimensions [8-10].

The aim of our study was to analyze the outcome in children with ALCAPA after cardiac surgery, especially with regard to LV systolic function, behavior, and progress of MV regurgitation during follow-up and somatic growth of children after surgical repair.

## Materials and methods

Retrospectively, we reviewed all children who underwent ALCAPA repair over 20 years (1999 - 2018).

Echocardiography (the sole imaging modality) reports were reviewed at the time of diagnosis, postoperatively, and during follow-up for ejection fraction (EF), fractional shortening (FS), LV inner diameter in diastole (LVIDD) z score, and LV inner diameter in systole (LVIDS) z score. These measurements were derived from the standard parasternal long-axis views [11]. The degree of MR was documented as per our echocardiography reports at the time of diagnosis and the last follow-up.

Demographic data: gender, age, and weight were reviewed. Immediate postoperative outcome data for intensive care unit (ICU) and hospital length of stay (LOS), morbidities, and mortality were evaluated as well. Weight centiles for the somatic growth were followed.

The echocardiographic data were stored and analyzed using Xcelera software (version 4.1; Philips, the Netherlands). The measurements were indexed on the basis of the built-in z score system in Xcelera.

Data are expressed as numbers and percentages for categorical variables and as mean ± standard deviation (SD) for continuous variables. Data that did not fit a normal distribution were expressed as median and interquartile range (IQR). The statistical analysis was performed using the Statistical Package for the Social Sciences (SPSS) software for Windows (version 22, IBM Corp, Armonk, NY). The study was approved by the Institutional Review Board (IRB) at our medical city.

## Results

During the study period, 29 patients underwent ALCAPA repair at the age of 5.3 (3.8-7.4) months and the weight of 5.5±2 kg. One patient presented late at the age of five years and was operated on successfully. Surgical repair was performed in a form of coronary reimplantation in 26 (90%) patients with MV repair in one of them and Takeuchi repair in three patients (10%).

ICU stay was eight (6-17) days and hospital stay was 15 (12-21) days. Duration of positive pressure ventilation (PPV) was two (1-4) days. Six patients (21%) had bloodstream infection (BSI) (Table 1). Five patients (17%) required readmission after discharge.

**Table 1:** Patients’ demographics and their postoperative course ALCAPA, anomalous origin of the left coronary artery from the pulmonary artery, ICU: intensive care unit; LOS, length of stay; PPV, positive pressure ventilation

Parameter	ALCAPA patients (n = 29)
Gender (female)	14 (48%)
Age at surgery (months)	5.3 (3.8 - 7.4)
Weight at surgery (kg)	5.5±2
Weight percentile at surgery (%)	6 ± 13
ICU LOS (days)	8 (6 - 17)
Hospital LOS (days)	15 (12 - 21)
PPV (days)	2 (1 - 4)
Bloodstream infection	6 (21%)
Surgical site infection	1 (3.4%)
Ventilator-associated pneumonia	2 (7%)

Associated cardiac lesions in patients with ALCAPA are shown in Table 2.

**Table 2:** Associated cardiac lesions in patients with ALCAPA ALCAPA, anomalous origin of the left coronary artery from the pulmonary artery

Associated lesions	Number of patients (%)
Mitral valve prolapse	3 (10%)
Atrial septal defect	4 (14%)
Aortopulmonary window	1 (3%)
Patent ductus arteriosus	4 (14%)

**Figure 1:**
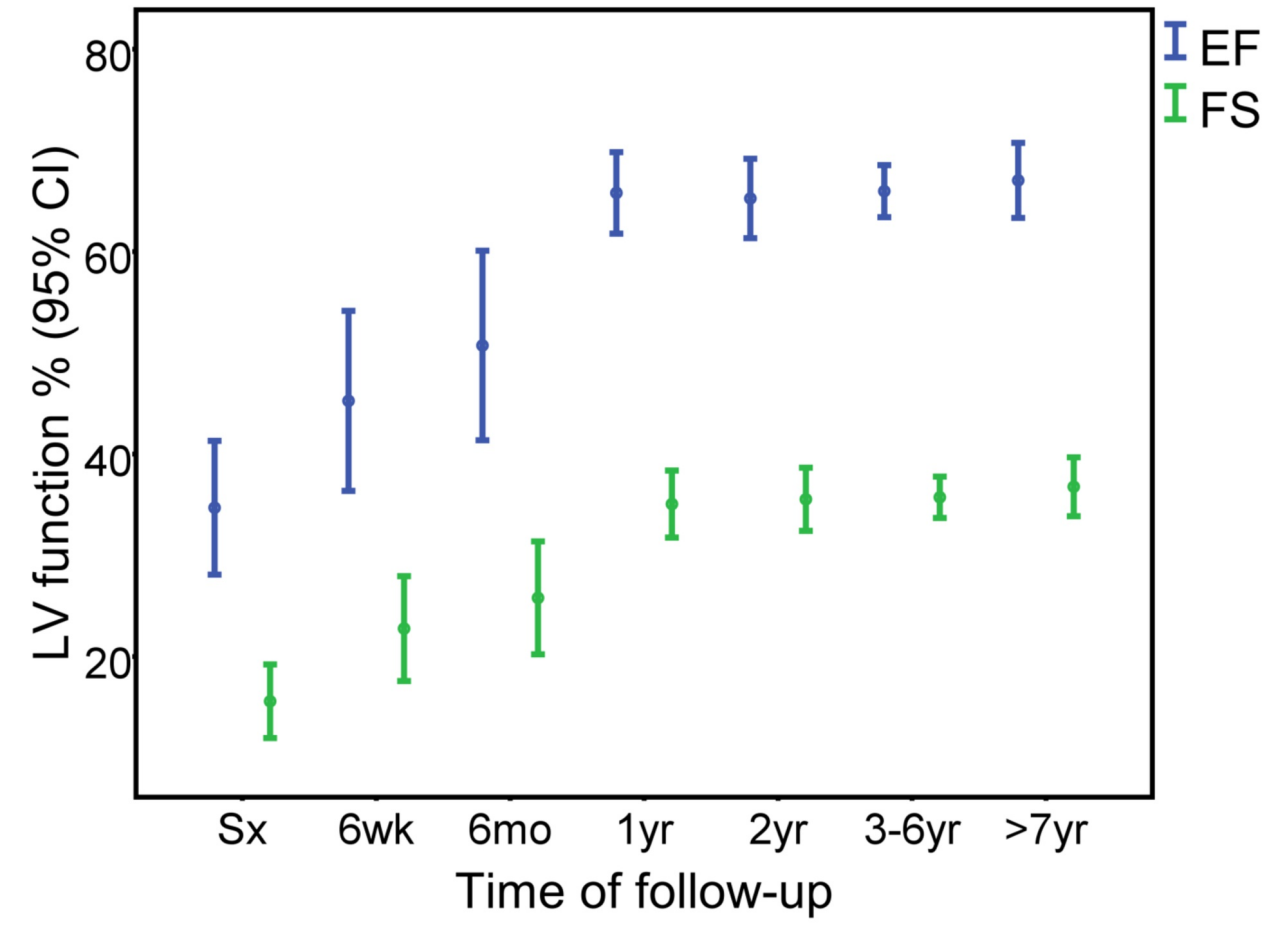
Ejection fraction (EF) and fractional shortening (FS) at different time intervals after surgery CI, confidence interval; Sx, surgery; mo, months; wk, weeks; yr, years

**Figure 2:**
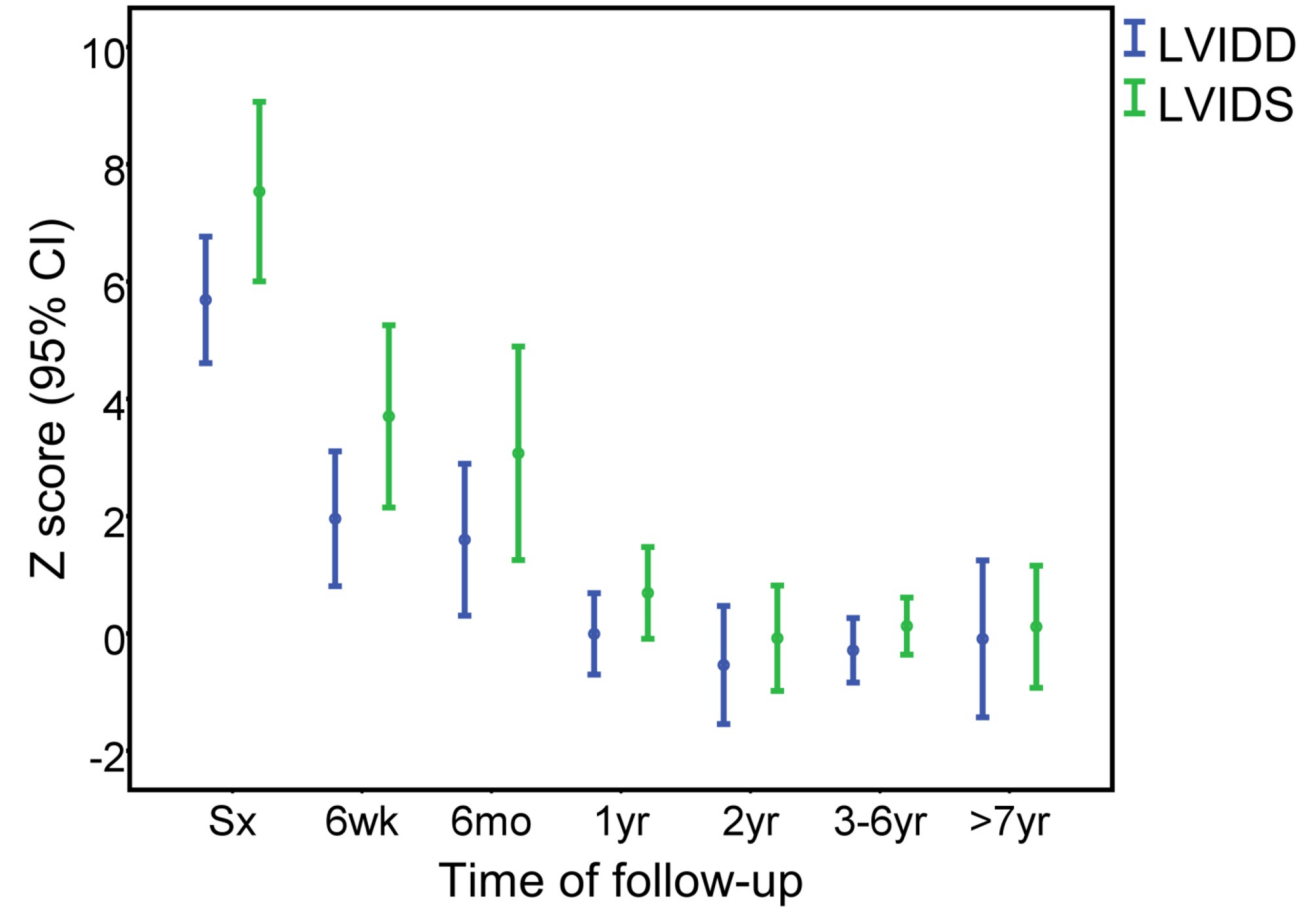
Left ventricular inner diameter during diastole (LVIDD) z score and left ventricular inner diameter during systole (LVIDS) z score at different time intervals after surgery CI, confidence interval; Sx, surgery; mo, months; wk, weeks; yr, years

**Figure 3:**
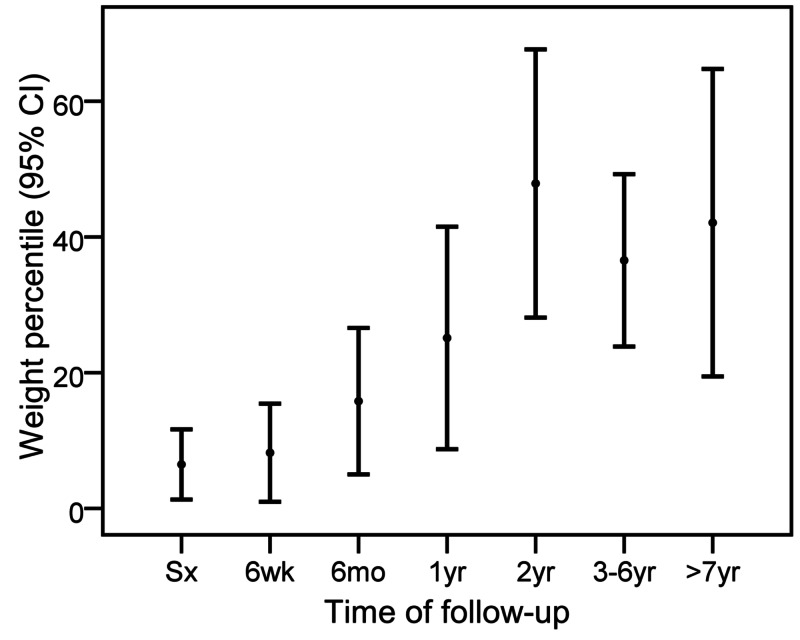
Weight for age percentile at different time intervals after surgery CI confidence interval; Sx, surgery; mo, months; wk, weeks; yr, years

**Figure 4:**
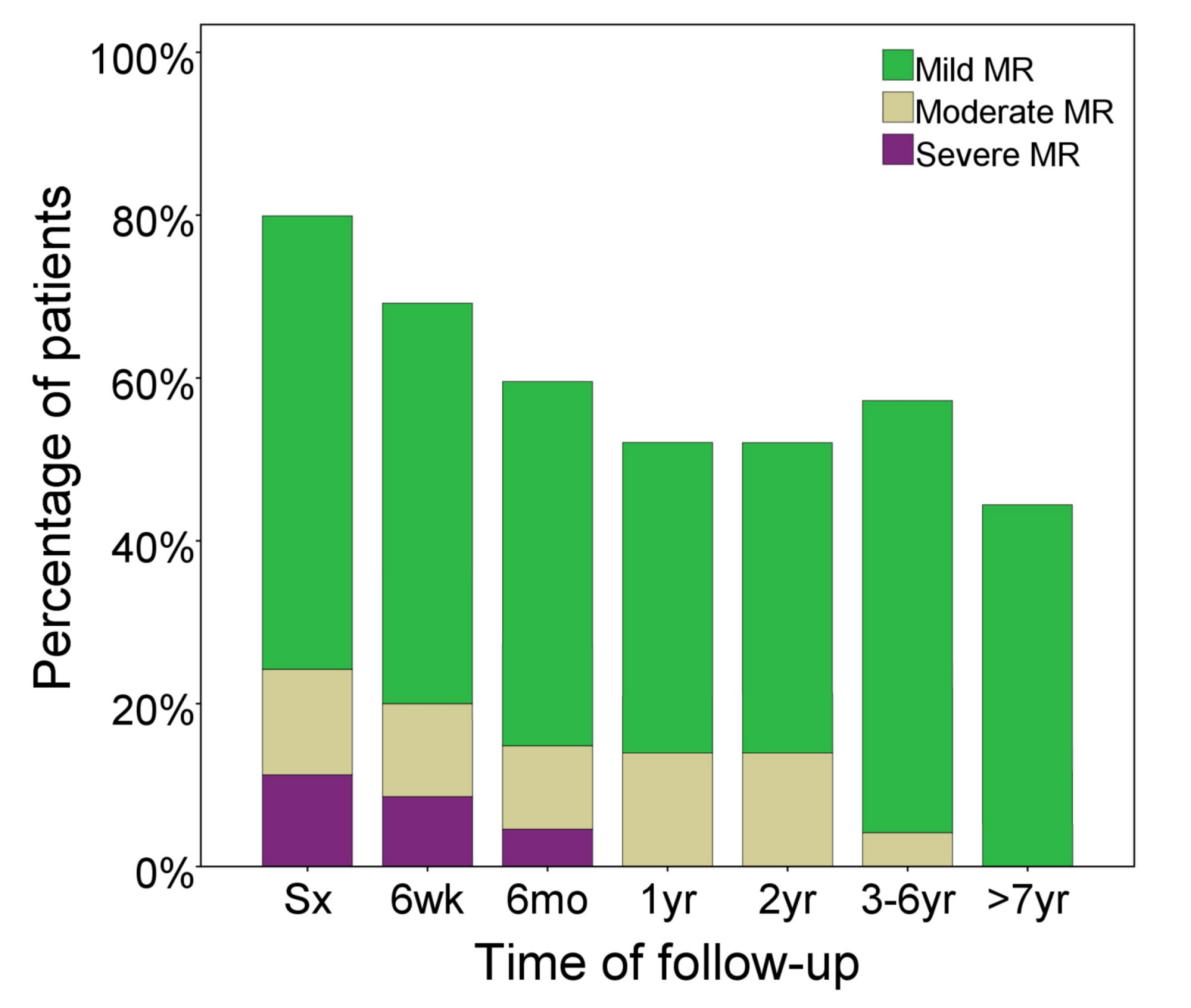
Spontaneous regression of MV regurgitation over the follow-up period MV, mitral valve; Mo, months; Sx, surgery; wk, weeks; yr, years

The follow-up duration was 5±3.6 years. Initial echocardiographic parameters for LV systolic function and dimensions were: EF 34±17%, FS 15±10%, and LVIDD z score 5.7±2.8. These parameters started to improve six weeks after surgery and reached normal ranges by one year after repair (Figure 1 and Figure 2). The initial weight percentile was 6±13%, which started to show improvement six months after surgical repair (Figure 3). Seven (24%) patients had moderate to severe MR at the time of surgery; however, there was no more moderate nor severe MR at the last follow-up (Figure 4). No patient required extracorporeal membrane oxygenation (ECMO) and there was no mortality.

## Discussion

ALCAPA is a rare coronary artery anomaly that requires a high index of suspicion. Echocardiography is the main imaging tool to establish the diagnosis while prompt surgical reestablishment of a two-coronary system after diagnosis leads to excellent results with better outcomes [6,12].

The majority of our patients (90%) were operated on by direct reimplantation of the anomalous coronary artery to the aorta, which appears to be the preferred method for many surgeons [6,8-9,13]. The remaining 10% were repaired through an intra-pulmonary baffle (known as Takeuchi procedure), which has its own complications in a form of supra-valvular pulmonary stenosis, baffle obstruction, baffle leaks, and aortic regurgitation [6,8]. However, none of our patients had any of those complications during the follow-up period. Similarly, Cabrera et al. reported that two (6%) out of their 34 patients required the Takeuchi procedure without complications [14].

The mean age and weight of our patient population at surgery were similar to the demographic results shown in other publications [13,15-16].

Serial postoperative echocardiography during follow-up showed complete recovery of LV systolic function in all our patients. LV systolic function started to improve six weeks after surgery and normalized at the latest one year after surgery. Simultaneously, the LVIDD z score decreased within the same period. Similar improvement in LV function was reported by Piotr et al. with 23 patients after ALCAPA repair [16]. In another series of 26 patients after ALCAPA repair, LV function normalized at a median time of five months (range: 0.5-36 months) and the LVIDD z score also decreased within the same period [15].

Only one of our patients (3%) required mechanical ventilation preoperatively, however, others reported that up to one-third of their patients were mechanically ventilated preoperatively [17]. None of the patients in our population required extracorporeal membrane oxygenation (ECMO), similar to what is reported in other papers [15,18].

ICU and hospital stay in our study were eight (6-17) days and 15 (12-21) days, respectively. Similar durations were reported earlier [4,7]. There was no mortality in our patients as documented by two other studies [18-19]. In some reports, however, there was higher mortality after ALCAPA repair (7%-17%) [15-17].

Somatic growth started to improve and normalized six months after surgery. To the best of our knowledge, there are no other studies that reported on somatic growth changes in this patient population after surgical repair of ALCAPA.

Whether to repair the MV at the same time as ALCAPA repair or not remains controversial. Several studies reported that simultaneous MV repair had no effect on the normalization of LV function or other surgical outcomes. They thought that with coronary revascularization, the recovery of LV dilatation and papillary dyskinesia could improve valve function [12-13,15-16,20-23]. Some recommended that simultaneous mitral annuloplasty should be performed at the time of ALCAPA repair in all patients with mild to severe MR [17,24].

The degree of MR improved gradually with the improvement of ventricular dimensions during the follow-up period. In our series, only one patient had MV repair at the time of initial surgery. Twenty-four percent (24%) of the rest of the patients had moderate to severe MR at diagnosis, which regressed to no more moderate nor severe MR at the last follow-up. Similarly, Cabrera et al. had 15 (44%) out of 34 patients with moderate to severe MR that decreased to 1 (5%) during follow-up [14].

None of our patients required reintervention during follow-up, in contrast to the results of Cabrera and his group; 15% of their patients required reintervention during follow-up, heart transplant in 6%, and MV replacement after initial valvuloplasty in 3% [14].

Limitations

This is a retrospective observational cohort study; thus, it is subject to all of the inherent bias that is inevitably related to this type of investigation. Further prospective studies of the LV functions by volumetric measures, speckle tracking, or perfusion scan may reveal a more accurate functional assessment. A multicenter study with a larger sample and a longer follow-up duration until adulthood may reveal long-term outcomes.

## Conclusions

LV systolic function and dimensions start to improve by six weeks after surgery and reach normal values by one year. MR regresses without intervention in correspondence with the regression of LV dimensional parameters. Children's somatic growth improves six months after repair.
